# 4-Nitro­benzoic acid–2,2′-biimidazole (2/1)

**DOI:** 10.1107/S1600536810015643

**Published:** 2010-05-08

**Authors:** Xin Liu, Weiqun Zhu

**Affiliations:** a School of Chemistry and Chemical Engineering, Shandong University, Jinan 250100, People’s Republic of China

## Abstract

In the title adduct, C_7_H_5_NO_4_·0.5C_6_H_6_N_4_, the complete biimidazole molecule is generated by a crystallographic inversion centre. In the crystal, N—H⋯O and O—H⋯N hydrogen bonds connects the 4-nitro­benzoic acid and 2,2′-biimidazole units, affording multi-dimensional frameworks with graph-set descriptor *R*_2_^2^(9).

## Related literature

For the potential applications of coordination complexes as functional materials and enzymes, see: Zhang *et al.* (2003 ▶) For hydrogen-bond motifs, see: Bernstein *et al.* (1995 ▶).
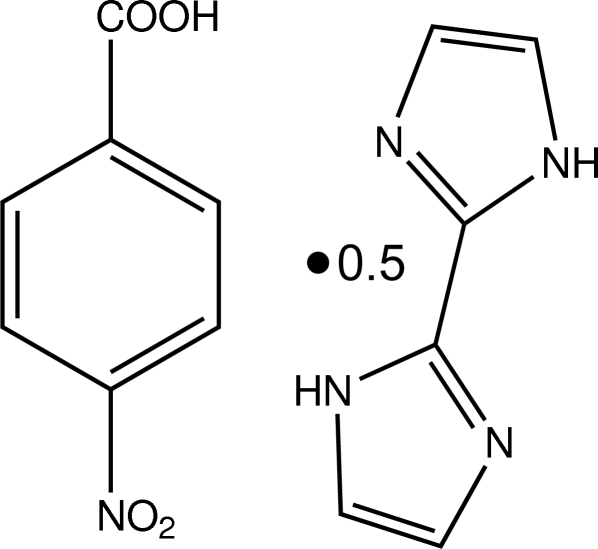


## Experimental

### 

#### Crystal data

C_7_H_5_NO_4_·0.5C_6_H_6_N_4_
*M* *_r_* = 234.19
Monoclinic, 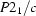

*a* = 4.852 (1) Å
*b* = 10.9245 (10) Å
*c* = 19.7981 (10) Å
β = 90.496 (1)°
*V* = 1049.4 (2) Å^3^
*Z* = 4
Mo *K*α radiation
μ = 0.12 mm^−1^
*T* = 296 K
0.12 × 0.10 × 0.08 mm

#### Data collection

Bruker APEXII CCD diffractometer
Absorption correction: multi-scan (*SADABS*; Bruker, 2001 ▶) *T*_min_ = 0.986, *T*_max_ = 0.991
5185 measured reflections
1849 independent reflections
1264 reflections with *I* > 2σ(*I*)
*R*_int_ = 0.034

#### Refinement

*R*[*F* ^2^ > 2σ(*F* ^2^)] = 0.045
*wR*(*F* ^2^) = 0.134
*S* = 1.00
1849 reflections
158 parameters
1 restraint
H atoms treated by a mixture of independent and constrained refinement
Δρ_max_ = 0.25 e Å^−3^
Δρ_min_ = −0.15 e Å^−3^

### 

Data collection: *APEX2* (Bruker, 2004 ▶); cell refinement: *SAINT-Plus* (Bruker, 2001 ▶); data reduction: *SAINT-Plus*; program(s) used to solve structure: *SHELXS97* (Sheldrick, 2008 ▶); program(s) used to refine structure: *SHELXL97* (Sheldrick, 2008 ▶); molecular graphics: *SHELXTL* (Sheldrick, 2008 ▶); software used to prepare material for publication: *SHELXTL*.

## Supplementary Material

Crystal structure: contains datablocks global, I. DOI: 10.1107/S1600536810015643/bx2278sup1.cif

Structure factors: contains datablocks I. DOI: 10.1107/S1600536810015643/bx2278Isup2.hkl

Additional supplementary materials:  crystallographic information; 3D view; checkCIF report

## Figures and Tables

**Table 1:** Hydrogen-bond geometry (Å, °)

*D*—H⋯*A*	*D*—H	H⋯*A*	*D*⋯*A*	*D*—H⋯*A*
O2—H2*A*⋯N1	0.85 (1)	1.75 (1)	2.580 (2)	168 (3)
N2—H2⋯O1^i^	0.86	1.89	2.742 (2)	173

Symmetry code: (i) 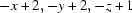.

**Fig. 1.**
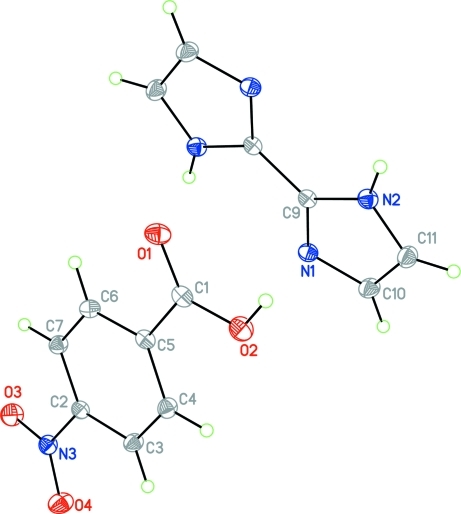
The molecular structure of (I), showing the atom-labelling scheme and with displacement ellipsoids at the 30% probability level. Unlabeled atoms are related to labeled atoms by the symmetry code (-x, 2-y, 1-z).

## supplementary crystallographic information

### Comment

Recently, the design and synthesis of coordination complexes have attracted much attention due to their diversity structures as well as potential applications as functional materials and enzymes (Zhang *et al.* , 2003). Here, we report one by-product of the hydrothermal reaction of FeCl_3_ with 4-nitrobenzoic acid and biimidazole. The asymmetric unit of (I) consists of a 4-nitrobenzoic acid molecule and half biimidazole molecule, Fig 1. In the 4-nitrobenzoic acid molecule, the nitro group is rotated 10.6 (3)° from aromatic ring. N—H···O and O—H···N hydrogen bonds connects the C_7_H_5_NO_4_ . 0.5C_6_H_6_N_4_ units to affords a macrocycle with graph-set descriptor R^2^_2_(9) (Bernstein *et al., 1995), Fig2.*

### Experimental

A mixture of 4-nitrobenzoic acid (1 mmoL, 0.17 g), biimidazole (1 mmoL, 0.14 g), and iron trichloride (1 mmoL, 0.27 g) in 12 ml distilled water sealed in a 25 ml Teflon-lined stainless steel autoclave was kept at 433 K for three days. Colorless crystals suitable for the single X-ray diffraction were obtained.

### Refinement

All H atoms were placed in calculated positions with C—H = 0.93Å and refined as riding with U_iso_(H) = 1.2U_eq_(carrier). The lengths of bond H—O were constrained with 0.82 Å .

### Crystal data

**Table:**

C_7_H_5_NO_4_·0.5C_6_H_6_N_4_	*F*(000) = 484
*M**_r_* = 234.19	*D*_x_ = 1.482 Mg m^−^^3^
Monoclinic, *P*2_1_/*c*	Mo *K*α radiation, λ = 0.71073 Å
Hall symbol: -P 2ybc	Cell parameters from 1230 reflections
*a* = 4.852 (1) Å	θ = 2.8–22.0°
*b* = 10.9245 (10) Å	µ = 0.12 mm^−^^1^
*c* = 19.7981 (10) Å	*T* = 296 K
β = 90.496 (1)°	Block, colourless
*V* = 1049.4 (2) Å^3^	0.12 × 0.10 × 0.08 mm
*Z* = 4	

### Data collection

**Table:**

Bruker APEXII CCD diffractometer	1849 independent reflections
Radiation source: fine-focus sealed tube	1264 reflections with *I* > 2σ(*I*)
graphite	*R*_int_ = 0.034
phi and ω scans	θ_max_ = 25.0°, θ_min_ = 2.1°
Absorption correction: multi-scan (*SADABS*; Bruker, 2001)	*h* = −5→5
*T*_min_ = 0.986, *T*_max_ = 0.991	*k* = −12→9
5185 measured reflections	*l* = −23→20

### Refinement

**Table:**

Refinement on *F*^2^	Secondary atom site location: difference Fourier map
Least-squares matrix: full	Hydrogen site location: inferred from neighbouring sites
*R*[*F*^2^ > 2σ(*F*^2^)] = 0.045	H atoms treated by a mixture of independent and constrained refinement
*wR*(*F*^2^) = 0.134	*w* = 1/[σ^2^(*F*_o_^2^) + (0.078*P*)^2^] where *P* = (*F*_o_^2^ + 2*F*_c_^2^)/3
*S* = 1.00	(Δ/σ)_max_ < 0.001
1849 reflections	Δρ_max_ = 0.25 e Å^−^^3^
158 parameters	Δρ_min_ = −0.15 e Å^−^^3^
1 restraint	Extinction correction: *SHELXL97* (Sheldrick, 2008), Fc^*^=kFc[1+0.001xFc^2^λ^3^/sin(2θ)]^-1/4^
Primary atom site location: structure-invariant direct methods	Extinction coefficient: 0.013 (4)

### Special details

**Table:**

Geometry. All esds (except the esd in the dihedral angle between two l.s. planes) are estimated using the full covariance matrix. The cell esds are taken into account individually in the estimation of esds in distances, angles and torsion angles; correlations between esds in cell parameters are only used when they are defined by crystal symmetry. An approximate (isotropic) treatment of cell esds is used for estimating esds involving l.s. planes.
Refinement. Refinement of F^2^ against ALL reflections. The weighted R-factor wR and goodness of fit S are based on F^2^, conventional R-factors R are based on F, with F set to zero for negative F^2^. The threshold expression of F^2^ > 2sigma(F^2^) is used only for calculating R-factors(gt) etc. and is not relevant to the choice of reflections for refinement. R-factors based on F^2^ are statistically about twice as large as those based on F, and R- factors based on ALL data will be even larger.

### Fractional atomic coordinates and isotropic or equivalent isotropic displacement parameters (Å^2^)

**Table:**

	*x*	*y*	*z*	*U*_iso_*/*U*_eq_	
C1	0.3475 (4)	0.7795 (2)	0.58109 (11)	0.0548 (6)	
C2	−0.2424 (4)	0.54924 (18)	0.67279 (9)	0.0493 (5)	
C3	−0.1097 (4)	0.5072 (2)	0.61681 (10)	0.0577 (6)	
H3	−0.1475	0.4298	0.5995	0.069*	
C4	0.0825 (4)	0.5826 (2)	0.58629 (11)	0.0586 (6)	
H4	0.1753	0.5557	0.5482	0.070*	
C5	0.1368 (4)	0.69760 (19)	0.61233 (10)	0.0502 (5)	
C6	−0.0063 (4)	0.7371 (2)	0.66845 (10)	0.0565 (6)	
H6	0.0283	0.8148	0.6858	0.068*	
C7	−0.1990 (4)	0.66362 (19)	0.69917 (11)	0.0551 (6)	
H7	−0.2963	0.6908	0.7366	0.066*	
C9	0.9965 (4)	0.95887 (18)	0.47185 (10)	0.0468 (5)	
C10	0.8856 (5)	0.8140 (2)	0.40287 (11)	0.0676 (7)	
H10	0.7961	0.7472	0.3836	0.081*	
C11	1.0980 (5)	0.8751 (2)	0.37532 (11)	0.0678 (7)	
H11	1.1813	0.8580	0.3342	0.081*	
H2A	0.566 (4)	0.7886 (19)	0.5083 (11)	0.080*	
N1	0.8218 (3)	0.86605 (16)	0.46396 (9)	0.0549 (5)	
N2	1.1672 (3)	0.96604 (16)	0.41881 (8)	0.0550 (5)	
H2	1.2968	1.0188	0.4135	0.066*	
N3	−0.4451 (4)	0.46869 (19)	0.70582 (9)	0.0596 (5)	
O1	0.4057 (3)	0.87721 (14)	0.60756 (8)	0.0689 (5)	
O2	0.4566 (3)	0.73838 (16)	0.52653 (8)	0.0739 (5)	
O3	−0.5946 (4)	0.50989 (16)	0.74887 (10)	0.0873 (6)	
O4	−0.4548 (4)	0.36262 (17)	0.68800 (10)	0.0926 (6)	

### Atomic displacement parameters (Å^2^)

**Table:**

	*U*^11^	*U*^22^	*U*^33^	*U*^12^	*U*^13^	*U*^23^
C1	0.0442 (12)	0.0579 (14)	0.0621 (14)	−0.0011 (10)	−0.0014 (10)	0.0114 (11)
C2	0.0449 (11)	0.0507 (12)	0.0522 (12)	−0.0036 (9)	0.0044 (9)	0.0051 (10)
C3	0.0628 (14)	0.0509 (12)	0.0595 (13)	−0.0100 (10)	0.0119 (11)	−0.0058 (10)
C4	0.0577 (14)	0.0624 (14)	0.0559 (13)	−0.0052 (11)	0.0124 (10)	−0.0028 (11)
C5	0.0427 (12)	0.0505 (13)	0.0574 (12)	−0.0028 (9)	−0.0024 (10)	0.0076 (10)
C6	0.0561 (13)	0.0481 (13)	0.0653 (13)	−0.0028 (10)	0.0007 (11)	−0.0026 (10)
C7	0.0543 (13)	0.0554 (13)	0.0556 (12)	0.0002 (10)	0.0060 (10)	−0.0031 (10)
C9	0.0399 (11)	0.0483 (12)	0.0523 (11)	0.0004 (9)	0.0024 (9)	0.0056 (9)
C10	0.0701 (16)	0.0621 (15)	0.0705 (15)	−0.0161 (12)	0.0037 (12)	−0.0095 (12)
C11	0.0719 (16)	0.0709 (16)	0.0609 (14)	−0.0068 (13)	0.0132 (12)	−0.0095 (13)
N1	0.0520 (11)	0.0516 (10)	0.0610 (11)	−0.0074 (8)	0.0014 (8)	0.0017 (9)
N2	0.0500 (10)	0.0544 (11)	0.0609 (11)	−0.0053 (8)	0.0088 (9)	0.0025 (9)
N3	0.0581 (12)	0.0634 (13)	0.0573 (11)	−0.0071 (10)	0.0092 (9)	0.0029 (9)
O1	0.0602 (10)	0.0563 (10)	0.0904 (11)	−0.0113 (8)	0.0095 (8)	0.0033 (9)
O2	0.0713 (12)	0.0737 (12)	0.0769 (11)	−0.0217 (9)	0.0177 (9)	0.0076 (9)
O3	0.0907 (13)	0.0871 (13)	0.0849 (12)	−0.0056 (10)	0.0419 (10)	0.0034 (10)
O4	0.1113 (16)	0.0649 (12)	0.1021 (13)	−0.0320 (10)	0.0356 (11)	−0.0089 (10)

### Geometric parameters (Å, °)

**Table:**

C1—O1	1.222 (3)	C7—H7	0.9300
C1—O2	1.288 (3)	C9—N1	1.330 (2)
C1—C5	1.496 (3)	C9—N2	1.345 (2)
C2—C3	1.366 (3)	C9—C9^i^	1.432 (4)
C2—C7	1.370 (3)	C10—C11	1.347 (3)
C2—N3	1.476 (3)	C10—N1	1.374 (3)
C3—C4	1.387 (3)	C10—H10	0.9300
C3—H3	0.9300	C11—N2	1.355 (3)
C4—C5	1.382 (3)	C11—H11	0.9300
C4—H4	0.9300	N2—H2	0.8600
C5—C6	1.384 (3)	N3—O3	1.211 (2)
C6—C7	1.377 (3)	N3—O4	1.212 (2)
C6—H6	0.9300	O2—H2A	0.845 (10)
			
O1—C1—O2	124.7 (2)	C2—C7—H7	121.1
O1—C1—C5	120.1 (2)	C6—C7—H7	121.1
O2—C1—C5	115.2 (2)	N1—C9—N2	110.41 (18)
C3—C2—C7	122.95 (19)	N1—C9—C9^i^	125.5 (2)
C3—C2—N3	118.65 (19)	N2—C9—C9^i^	124.1 (2)
C7—C2—N3	118.40 (18)	C11—C10—N1	109.3 (2)
C2—C3—C4	118.5 (2)	C11—C10—H10	125.3
C2—C3—H3	120.7	N1—C10—H10	125.3
C4—C3—H3	120.7	C10—C11—N2	106.98 (19)
C5—C4—C3	120.3 (2)	C10—C11—H11	126.5
C5—C4—H4	119.9	N2—C11—H11	126.5
C3—C4—H4	119.9	C9—N1—C10	105.70 (18)
C4—C5—C6	119.17 (19)	C9—N2—C11	107.60 (17)
C4—C5—C1	121.2 (2)	C9—N2—H2	126.2
C6—C5—C1	119.7 (2)	C11—N2—H2	126.2
C7—C6—C5	121.3 (2)	O3—N3—O4	122.56 (19)
C7—C6—H6	119.3	O3—N3—C2	119.7 (2)
C5—C6—H6	119.3	O4—N3—C2	117.72 (18)
C2—C7—C6	117.76 (19)	C1—O2—H2A	113.3 (17)
			
C7—C2—C3—C4	1.6 (3)	C5—C6—C7—C2	0.7 (3)
N3—C2—C3—C4	−179.36 (18)	N1—C10—C11—N2	−0.4 (3)
C2—C3—C4—C5	−0.1 (3)	N2—C9—N1—C10	−0.6 (2)
C3—C4—C5—C6	−1.1 (3)	C9^i^—C9—N1—C10	178.9 (2)
C3—C4—C5—C1	178.72 (18)	C11—C10—N1—C9	0.6 (3)
O1—C1—C5—C4	−175.01 (18)	N1—C9—N2—C11	0.4 (2)
O2—C1—C5—C4	4.7 (3)	C9^i^—C9—N2—C11	−179.1 (2)
O1—C1—C5—C6	4.8 (3)	C10—C11—N2—C9	0.0 (2)
O2—C1—C5—C6	−175.56 (18)	C3—C2—N3—O3	−168.9 (2)
C4—C5—C6—C7	0.7 (3)	C7—C2—N3—O3	10.2 (3)
C1—C5—C6—C7	−179.06 (18)	C3—C2—N3—O4	10.8 (3)
C3—C2—C7—C6	−1.9 (3)	C7—C2—N3—O4	−170.2 (2)
N3—C2—C7—C6	179.04 (18)		

Symmetry codes: (i) −*x*+2, −*y*+2, −*z*+1.

### Hydrogen-bond geometry (Å, °)

**Table:**

*D*—H···*A*	*D*—H	H···*A*	*D*···*A*	*D*—H···*A*
O2—H2A···N1	0.85 (1)	1.75 (1)	2.580 (2)	168 (3)
N2—H2···O1^i^	0.86	1.89	2.742 (2)	173

Symmetry codes: (i) −*x*+2, −*y*+2, −*z*+1.
